# Access to Thrombolysis for Non-Resident and Resident Stroke Patients—A Registry-Based Comparative Study from Berlin

**DOI:** 10.3389/fneur.2017.00319

**Published:** 2017-06-30

**Authors:** Ludwig Schlemm, Guillaume Turc, Heinrich J. Audebert, Martin Ebinger

**Affiliations:** ^1^Department of Neurology, Charité – Universitätsmedizin Berlin, Berlin, Germany; ^2^Center for Stroke Research Berlin (CSB), Charité – Universitätsmedizin, Berlin, Germany; ^3^Berlin Institute of Health (BIH), Berlin, Germany; ^4^London School of Economics and Political Science, London, United Kingdom; ^5^Department of Neurology, Hôpital Sainte-Anne, Paris, France; ^6^INSERM U894, Paris, France; ^7^Department of Neurology, MEDICAL PARK Berlin Humboldtmühle, Berlin, Germany

**Keywords:** acute ischemic stroke, cerebrovascular diseases, epidemiology of stroke, health services, thrombolysis, emergency medical services

## Abstract

**Objectives:**

Stroke can happen to people away from home. It is unknown whether non-resident and resident stroke patients have equal access to thrombolysis.

**Materials and methods:**

Consecutive patients cared for by the Stroke Emergency Mobile between 2011 and 2016 after prompting suspicion of acute stroke during the emergency call were included in our registry. Patients were categorized as residents or non-residents based on their main address. Clinical characteristics, thrombolysis rates, and time intervals from symptom onset/last seen well to alarm and to thrombolysis were compared between groups adjusting for age, pre-stroke modified Rankin Scale (mRS) score, and National Institutes of Health Stroke Scale (NIHSS) score.

**Results:**

Of 4,254 patients for whom a stroke dispatch was activated, 2,451 had ischemic or hemorrhagic strokes, including 73 non-residents. Non-resident stroke patients were younger (median 69.4 vs. 76.6 years, *p* < 0.001), had less pre-stroke disability (mRS ≥ 2:17.8 vs. 47.5%, *p* < 0.001) and less severe strokes (median NIHSS 4 vs. 5, *p* = 0.02). Thrombolysis rates were higher in non-residents (30.9 vs. 22.0% of ischemic stroke patients, *p* = 0.04) and emergency calls were made faster (symptom onset/last-seen-well-to-alarm time 35 vs. 144 min, *p* = 0.04). A lower proportion of non-residents had unknown time of symptom onset (21.9 vs. 46.4%, *p* < 0.001). For patients with known time of symptom onset, thrombolysis rates, and prehospital delays were similar among non-residents and residents.

**Conclusion:**

In this study, non-resident stroke patients had higher rates of thrombolysis than residents. This may be explained by a lower proportion of patients with unknown time of symptom onset.

## Introduction

Mobility and traveling at the international level has been increasing constantly at a rate of approximately 3.9% annually (1). For the years 2011–2015, approximately 1.2 billion tourist trips of residents were reported for the European Union each year, of which approximately 75% were domestic (2, 3). In the European Union, between 17 and 18% of tourists were older than 65 years (4, 5). As the general population is getting older (6), the number of elderly tourists is likely to increase in the future. People in this age group tend to have more premorbid conditions (7) and may be more likely to need medical attention while away from their homes. Stroke and transient ischemic attack are common among tourist patients presenting to emergency departments (8) and correspond to the most frequent medical conditions leading to repatriation (9). Immediate medical care and treatment are of crucial importance for patients with suspected stroke and lead to improved outcome (10, 11). It remains uncertain whether staying in a foreign city affects the decision-making of individuals with stroke symptoms or witnesses to call emergency services and patients’ access to timely treatment. We aimed to compare clinical characteristics, rates of intravenous thrombolysis, and prehospital delays between non-residents and residents stroke patients in a large prehospital registry.

## Materials and Methods

### Study Design

We conducted a retrospective cross-sectional study in Berlin, Germany. Consecutive adult patients managed by a specialized prehospital stroke ambulance equipped with a computerized tomography (CT) scanner and staffed with a stroke neurologist [the Stroke Emergency Mobile (STEMO)] between February 2011 and November 2016 were entered into a prospective registry, as described previously (12–14). Briefly, STEMO covered an area of 1.3 million inhabitants and was deployed if the emergency call center activated a stroke dispatch with assumed time from symptom onset less than 4 h or with unknown time from symptom onset. The STEMO was available 7 days a week between 7:00 a.m. and 11:00 p.m. during the time between May 9, 2011 and May 26, 2013; otherwise, STEMO could be dispatched between 7:00 a.m. and 7:00 p.m. A first-response ambulance was dispatched simultaneously and was able to cancel STEMO based on their assessment. After patients were seen by the stroke neurologist on board of STEMO and had obtained a prehospital non-contrast head-CT if necessary, they were given a diagnosis of ischemic stroke/transient ischemic attack, hemorrhagic stroke, or other (stroke mimic). The final determination of the time of symptom onset was made by the stroke neurologist on scene. If indicated, intravenous thrombolysis with alteplase was started on STEMO within 4.5 h of symptom onset.

Patients were eligible for the present study if a valid address was available; the main analysis was restricted to patients with a diagnosis of stroke. We categorized patients as residents if they lived within the boundaries of the state of Berlin as judged by their main address; otherwise patients were categorized as non-residents. The following data were prospectively collected in the registry: age, gender, diagnosis, presence of pre-stroke disability [modified Rankin Scale (mRS) ≥2], severity of stroke symptoms [National Institutes of Health Stroke Scale (NIHSS) score], time of symptom onset, time last seen well (LSW), time of STEMO arrival, time of CT-scan, and time of thrombolysis in eligible patients. For residents and non-residents, the proportion of patients with known time of symptom onset was recorded. In order to calculate time intervals, time of symptom onset was replaced by time LSW if the former was not known. Patients for whom neither time of symptom onset nor time LSW was available, or for whom time data were inconsistent, were excluded from the analysis of time intervals The primary endpoint of our study was the difference in the proportion of ischemic stroke patients receiving thrombolysis between non-residents and residents after adjustment for potential confounders. Secondary endpoints were symptom-onset/LSW-to-alarm time, symptom-onset/LSW-to-STEMO arrival time, and the proportion of all stroke patients for whom the emergency call was made within 4.5 h.

### Statistical Analysis

Continuous data were summarized by the median and interquartile range. Categorical data were summarized by absolute numbers and percentages. Crude differences of distributions between the non-resident and resident groups were assessed by independent samples Mann–Whitney *U* tests or Pearson’s chi-squared tests, as appropriate. Differences after adjustment for potential confounders were assessed by analysis of covariance and binomial logistic regression models. Temporal variables were positively skewed and underwent logarithmic transformation before entering analyses of variance as dependent variable. For comparisons involving thrombolysis and prehospital delay times, reported *p*-values were adjusted for potential confounders who were distributed differently among non-residents and residents: age, NIHSS score, and pre-stroke disability (mRS ≥ 2). A two-sided *p*-value of less than 0.05 was considered statistically significant. Data were analyzed with IBM SPSS Statistics version 21 (IBM Corp., Armonk, NY, USA) and MATLAB (Mathworks, Inc.).

## Results

**Figure 1:**
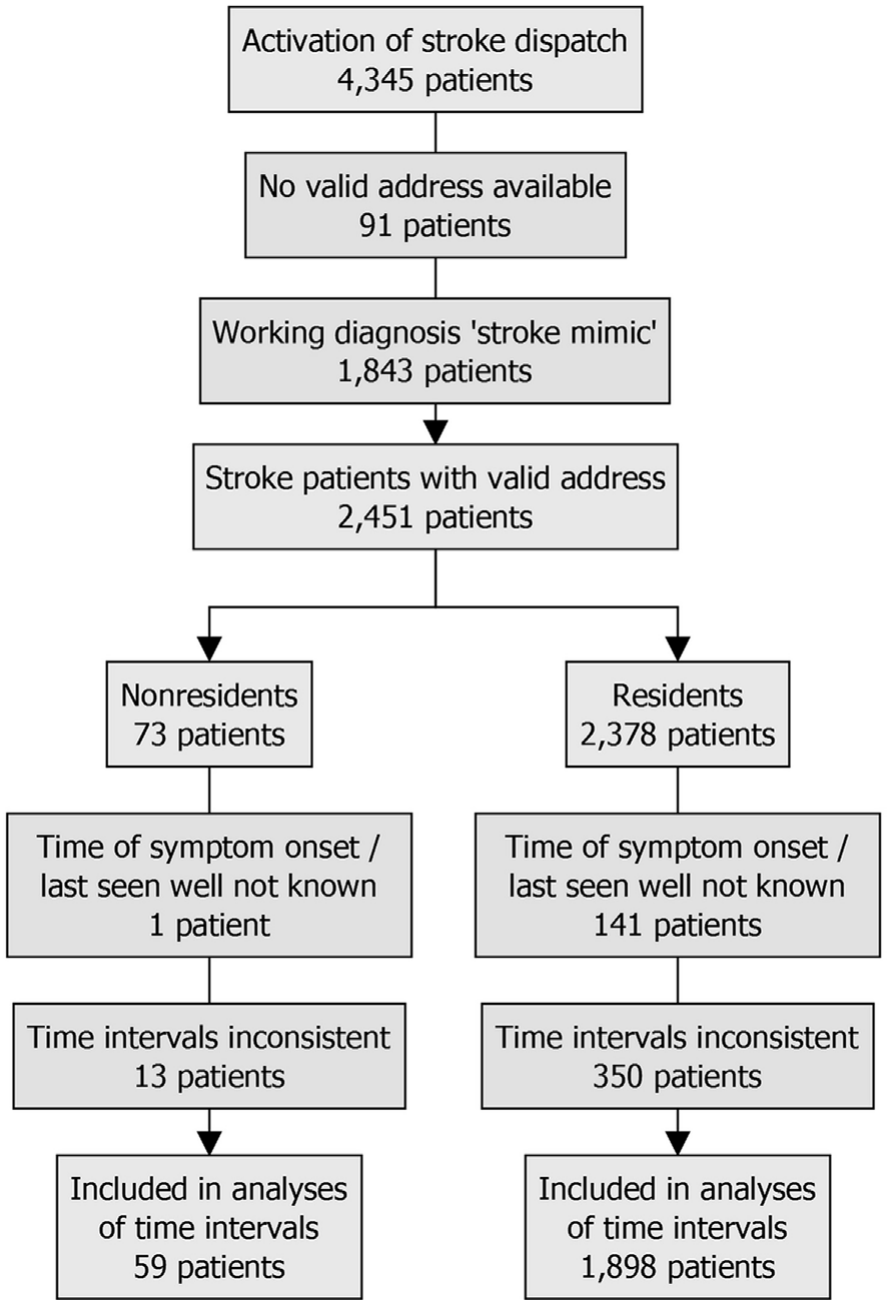
Flowchart.

**Figure 2:**
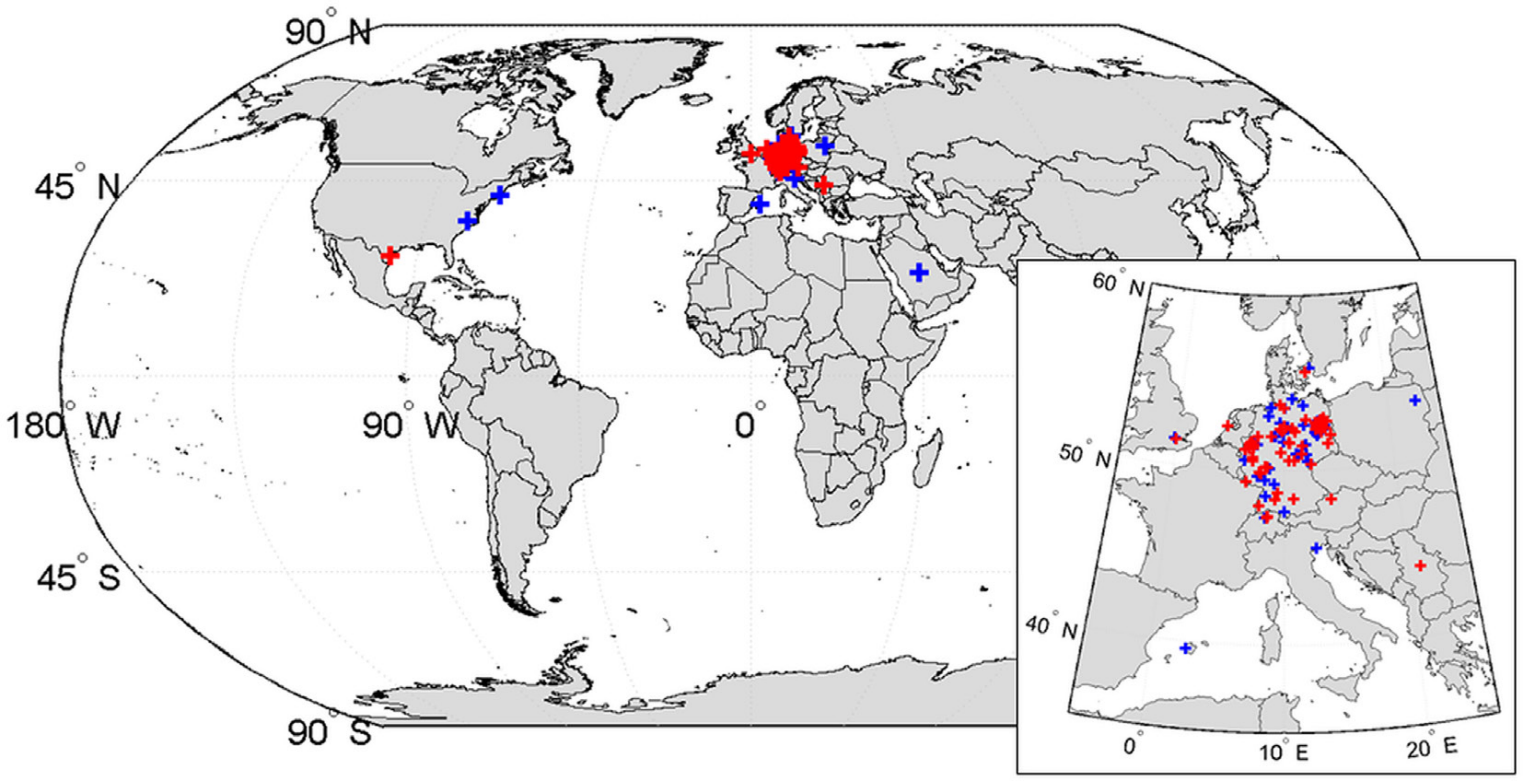
Geographical distribution of non-residents’ principal residences. The geographical locations of principal residences of non-resident patients with suspected stroke are displayed. Red crosses represent patients with a diagnosis stroke (73 patients), blue crosses stroke mimic (58 patients). The principal residence of most patients (114 of 131) was in Germany. Four patients had their principal residence outside of Europe: three patients were residents of the United States, one patient was a resident of Saudi Arabia. World borders dataset used to create the map from http://thematicmapping.org, available under a Creative Commons Attribution-Share Alike License (15).

The flowchart of the study is presented in Figure 1. A total of 4,345 consecutive patients were included in our prehospital registry. Ninety-one (2.1%) patients were excluded from further analysis because no valid address was available. Of the remaining 4,254 patients, 131 (3.1%) were non-residents. Forty-five (34.4%) lived in the state of Brandenburg, which surrounds the state of Berlin, and 17 (13.0%) came from abroad. The median distance between non-residents’ main address and the city center of Berlin was 241 (31–468) km (Figure 2) (15). Compared with the resident group, the proportions of non-residents diagnosed with ischemic stroke [68 (51.9%) vs. 2,259 (54.8%)], hemorrhagic stroke [5 (3.8%) vs. 119 (2.9%)], and stroke mimics [56 (42.7%) vs. 1,694 (41.1%)] were similar (*p* = 0.72 for comparison).

**Figure 3:**
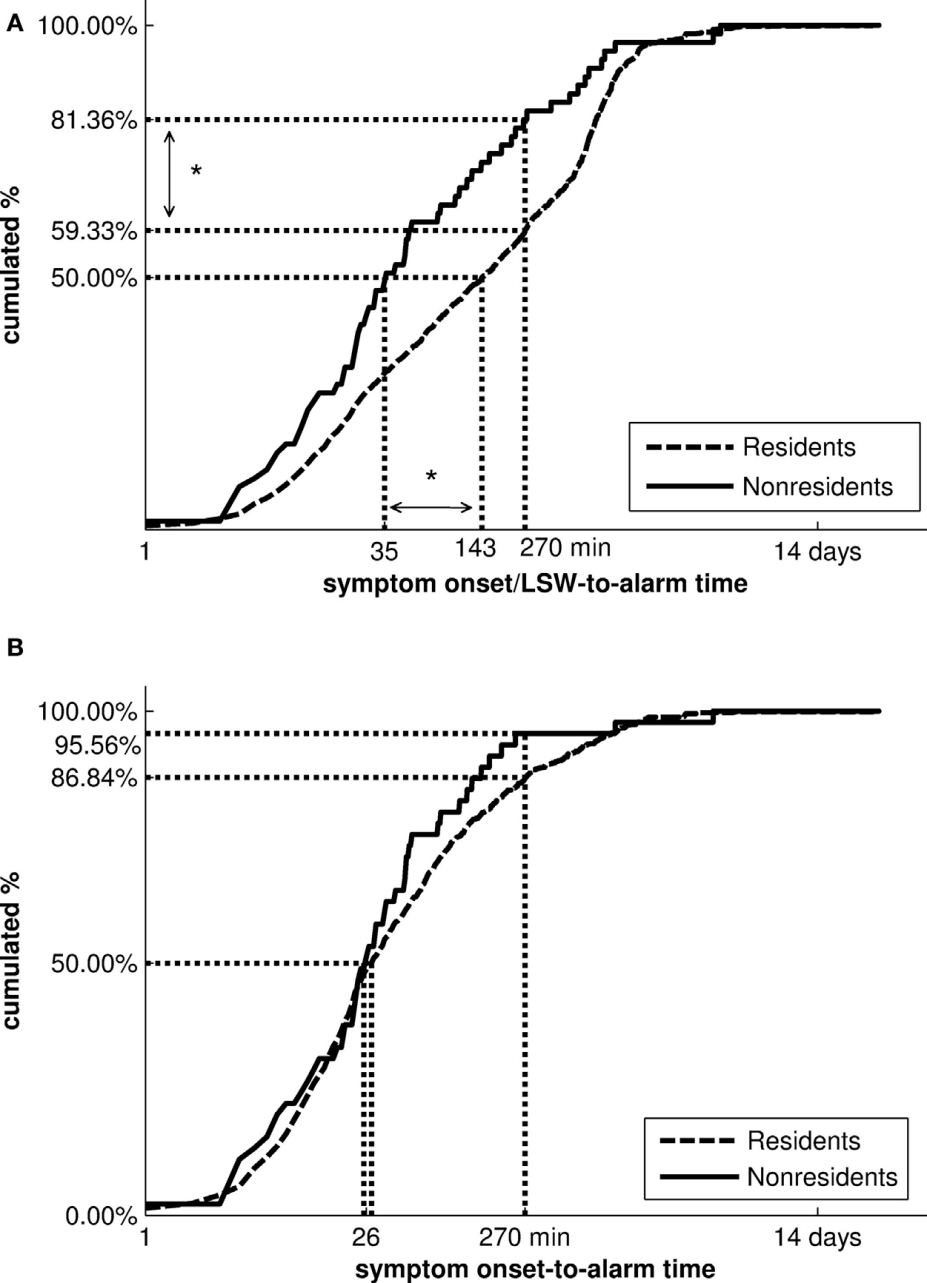
Cumulative distributions of symptom onset/LSW-to-alarm times and symptom onset-to-alarm times of non-resident and resident stroke patients. Vertical and horizontal dotted lines visualize the distributions’ median and the proportion of patients with a symptom onset/LSW-to-alarm and symptom onset-to-alarm time of less than 4.5 h. Note the logarithmic time scale on the horizontal axis. **(A)** The distribution of symptom onset/LSW-to-alarm times was significantly different between non-residents and residents with a diagnosis of stroke after adjustment for confounders (age, modified Rankin Scale Score, National Institutes of Health Stroke Scale score). **(B)** In patients with known time of symptom onset, no differences in the symptom onset-to-alarm times were observed (*p < 0.05). LSW, last seen well.

Among 2,451 patients with a diagnosis of stroke, 73 (3.0%) were non-residents. Non-resident stroke patients were significantly younger than residents (69.4 vs. 76.6 years; *p* < 0.001), had lower rates of pre-stroke disability (mRS ≥ 2:17.8 vs. 47.5%, *p* < 0.001), and had severe stroke symptoms at first assessment (median NIHSS score 4 vs. 5; *p* = 0.02; Table 1). Median symptom onset/LSW-to-alarm time and symptom onset/LSW-to-STEMO arrival time were significantly shorter in non-resident stroke patients than in residents (35 vs. 144 min, *p* = 0.04; and 62 vs. 179 min, *p* = 0.02, respectively; Table 2). The proportion of emergency calls made within 4.5 h after symptom-onset/LSW was higher for non-residents than for residents [81.4 vs. 59.3%, odds ratio = 2.11, 95% Confidence Interval (CI): 1.07–4.18, *p* = 0.03; Figure 3A].

**Table 1:** Baseline characteristics of non-resident and resident stroke patients.

	Non-residents	Residents	*p*-value^a^
Number of patients, *n*	73	2,378	–
Age (years); median (IQR)	69.4 (58.1–78.1)	76.6 (68.7–84.3)	<0.001
Women, *n* (%)	31 (42.5%)	1,256 (52.8%)	0.081
Distance to center of Berlin (km), median (IQR)	237 (36–462)	8 (5–10)	<0.001
Pre-stroke mRS score, median (IQR)	0 (0–1)	1 (0–3)	<0.001
Pre-stroke mRS score, *n* (%)^b^			
≤1	57 (78.1%)	1,216 (51.1%)	<0.001
≥2	13 (17.8%)	1,130 (47.5%)	
Missing	3 (4.1%)	32 (1.3%)	
NIHSS score, median (IQR)	4 (1–7)	5 (2–10)	0.02
Unknown time of symptom onset, *n* (%)	16 (21.9%)	1,103 (46.4%)	<0.001

*^a^Unadjusted p-values*.

*^b^Columns may not sum to 100% because of rounding*.

*IQR, interquartile range; mRS, modified Rankin Scale; NIHSS, National Institutes of Health Stroke Scale*.

**Table 2:** Distribution of time intervals for non-resident and resident stroke patients.

	Non-residents		Residents		
Time interval	*n*_crude_ *n*_adj_	Median (IQR)	*n*_crude_ *n*_adj_	Median (IQR)	*p*_crude_ *p*_adj_
Onset-to-alarm	59 50	35 (12–191)	1,898 1,783	144 (23–658)	<0.001 0.04
Onset-to-stroke emergency mobile (STEMO) arrival	59 50	62 (41–224)	1,898 1,783	179 (54–690)	<0.001 0.02
Onset-to-CT	31 29	70 (44–257)	850 835	121 (60–588)	0.15 0.18
Onset-to-thrombolysis	13 13	71 (48–86)	331 324	88 (61–140)	0.13 0.22
STEMO arrival-to-CT	31 29	12 (8–39)	850 835	14 (9–33)	0.32 0.74
CT-to-thrombolysis	13 13	9 (8–12)	331 324	10 (7–16)	0.50 0.52

*Displayed are number of available cases (*n*_crude_ and *n*_adj_, which takes into account availability of potential confounders) and times in minutes [median and interquartile range (IQR)]. *p*-values are reported for univariable (unadjusted) und multivariable (adjusted for age, National Institutes of Health Stroke Scale score, and modified Rankin Scale Score) analyses. CT, computerized tomography*.

A higher proportion of non-resident ischemic stroke patients received thrombolysis than residents [21 (30.9%) of 68 vs. 497 (22.0%) of 2,259 patients]. After adjustment for age, preexisting disability, and stroke severity, non-residents with ischemic stroke had a significantly higher probability to receive thrombolysis (odds ratio = 1.93, 95% CI: 1.09–3.42, *p* = 0.04; unadjusted odds ratio = 1.59, 95% CI: 0.94–2.68, *p* = 0.08). The median time between symptom onset and thrombolysis was not significantly different between non-residents and residents (71 vs. 88 min, *p* = 0.22).

In 21.9% of non-residents and 46.4% of residents with stroke (*p* < 0.001), time of symptom onset was not available, and it was unknown whether onset was within the 4.5-h time window for thrombolysis. This difference remained significant after adjustment for age, NIHSS score, and pre-stroke disability (*p* = 0.02). In the subgroup of patients with known time of symptom onset, delays in making the emergency call did not differ significantly between non-resident and resident stroke patients (Figure 3B). Also, the proportion of patients receiving thrombolysis was similar for non-resident and resident ischemic stroke patients with known symptom onset (35.2 vs. 31.5%; *p* = 0.28).

## Discussion

We compared clinical characteristics and prehospital management between 73 non-resident and 2,378 resident stroke patients in Berlin, for whom a stroke dispatch was activated. The main findings of our study are that, compared to residents, non-residents (1) were on average 7 years younger, had a lower burden of pre-stroke disability and less severe strokes; (2) had higher thrombolysis rates after adjustment for age, mRS, and NIHSS; and (3) had a significantly shorter symptom onset/LSW-to-alarm time.

The different patient characteristics can be explained by a lower proportion of people aged over 65 to engage in tourism (4, 5) or to commute, and the observation that older age is associated with stroke severity (16) and a higher probability of having had a prior stroke. Additionally, due to reduced mobility, people with pre-stroke disability might be less likely to travel, irrespective of their age.

With regard to prehospital stroke management, the median difference of symptom onset/LSW-to-alarm times between both groups was 109 min in our cohort. Accordingly, non-resident stroke patients were seen by the prehospital stroke team earlier after symptom onset than residents and had an almost twofold higher odds of receiving thrombolysis. Previous studies have shown that younger age and higher NIHSS score are associated with shorter prehospital delays (17–20) and non-residents were younger than residents in our study. Indeed, considering the significance levels of our results obtained with and without adjustment, the between-group differences of symptom onset/LSW-to alarm and symptom onset/LSW-to-treatment time are partly, but not fully, explained by age, stroke severity, and pre-stroke disability. More residents than non-residents had an unknown time of symptom onset in our study, which explains the residual between-group difference. This imbalance was not only due to differences in age and stroke severity (e.g., inability to respond to questions due to motor aphasia or reduced level of consciousness) but also might be reflective of the fact that non-residents have a higher level of planned activities and more witnessed time while away from home. Especially since our study was mainly conducted during daytime (7:00 a.m.–11:00 p.m.), non-residents can be expected to be often either at work or visiting Berlin. Additional factors that have previously been found to be associated with prehospital delay and that are likely to be distributed unevenly between non-residents and residents are socioeconomic status (21, 22) and the tendency to contact a general practitioner when stroke symptoms occur (23). We did not have the necessary data to explore the influence of these parameters in more detail.

The proportion of strokes with unknown time of symptom onset in our study was 21.9% for non-residents and 46.4% for residents. Previous studies have reported rates of approximately 30% (24–28). Determination of time of symptom onset sometimes requires time for additional phone calls with patients’ relatives and caregivers whose contact details may not be immediately known. Therefore, the fact that our results are solely based on prehospital data may explain the slightly higher rate of stroke with unknown time of symptom onset in our cohort.

In agreement with the observation that most touristic trips in Europe are domestic (3), the great majority of non-resident patients (114 of 131) included in our registry lived in Germany. Therefore, factors that could become relevant in the context of international travel, such as language barriers, unfamiliarity with the local health-care system, and reluctance to seek medical assistance due to lack of financial resources and/or health insurance, are less likely to have influenced our results. Additionally, for residents of Germany, health insurance is mandatory and emergency medical care is free of charge at the point of service (29). It has been estimated that comprehensive health insurance increases utilization of health-care services (30, 31). As a consequence, our results may not be generalizable to areas with higher proportions of international tourists or different health-care systems.

Some limitations have to be considered when interpreting our findings. First, in the subgroup of patients with known symptom onset time, our study was limited by low statistical power (high risk of type II error). Indeed, *post hoc* calculations in this subgroup suggested a power of respectively 10 and 11% to obtain statistically significant results in the comparisons of onset-to-alarm times and proportions of thrombolysis between non-residents and residents at a two-sided alpha level of 0.05. This low observed statistical power can not only be explained by the small number of patients in the non-resident group and the greatly unequal sample sizes (32) but also by the small between-group differences (thrombolysis rates: 35.2 vs. 31.5%, median onset-to-alarm time: 25 vs. 26 min). Second, we did not include information on vascular risk factors and comorbidities, which may be distributed differentially between non-residents and residents. Third, a full set of time intervals was missing for approximately 20% of all patients; however, proportions of missing data were similar for non-residents and residents, and a non-random distribution of missing items between the two groups, which could have introduced bias (differential misclassification), seemed unlikely based on our study design. Fourth, we only included patients with suspected stroke for whom an ambulance call was made between 7:00 a.m. and 11:00 p.m. that were seen by the STEMO team; therefore, our results do not necessarily apply to all patients making an emergency call with symptoms suggesting an acute stroke.

## Conclusion

In summary, non-residents had a higher thrombolysis rate and shorter symptom onset/LSW-to-alarm time compared to Berlin residents. These results can be explained by a lower proportion of patients with unknown time of symptom onset among non-residents, probably reflecting more witnessed stroke events during the daytime.

## Ethics Statement

This study was carried out in accordance with the recommendations of the local ethics committee at Charité – Universitätsmedizin Berlin. Written informed consent of all patients or legally authorized representatives for data use was obtained in STEMO or as soon as possible during in-hospital stay. The protocol was approved by the local ethics committee at Charité – Universitätsmedizin Berlin.

## Author Contributions

LS researched the literature, conceived the study, performed the data analysis, interpreted the data, and wrote the first draft of the manuscript. ME was involved in conceiving the study, patient recruitment, and interpretation of the data. GT made substantial contributions to the study design, data analysis, and interpretation of the data. HA was involved in patient recruitment and interpretation of the data. All authors critically reviewed and edited the manuscript and approved the final version of the manuscript.

## Conflict of Interest Statement

The authors declare that the research was conducted in the absence of any commercial or financial relationships that could be construed as a potential conflict of interest.
